# Replicative homeostasis II: Influence of polymerase fidelity on RNA virus quasispecies biology: Implications for immune recognition, viral autoimmunity and other "virus receptor" diseases

**DOI:** 10.1186/1743-422X-2-70

**Published:** 2005-08-22

**Authors:** Richard Sallie

**Affiliations:** 1 Suite 35, 95 Monash Avenue, Nedlands, Western Australia, 6009, Australia

## Abstract

Much of the worlds' population is in active or imminent danger from established infectious pathogens, while sporadic and pandemic infections by these and emerging agents threaten everyone. RNA polymerases (RNA_pol_) generate enormous genetic and consequent antigenic heterogeneity permitting both viruses and cellular pathogens to evade host defences. Thus, RNA_pol _causes more morbidity and premature mortality than any other molecule. The extraordinary genetic heterogeneity defining viral quasispecies results from RNA_pol _infidelity causing rapid cumulative genomic RNA mutation a process that, if uncontrolled, would cause catastrophic loss of sequence integrity and inexorable quasispecies extinction. Selective replication and replicative homeostasis, an epicyclical regulatory mechanism dynamically linking RNApol fidelity and processivity with quasispecies phenotypic diversity, modulating polymerase fidelity and, hence, controlling quasispecies behaviour, prevents this happening and also mediates immune escape. Perhaps more importantly, ineluctable generation of broad phenotypic diversity after viral RNA is translated to protein quasispecies suggests a mechanism of disease that specifically targets, and functionally disrupts, the host cell surface molecules – including hormone, lipid, cell signalling or neurotransmitter receptors – that viruses co-opt for cell entry. This mechanism – "Viral Receptor Disease (VRD)" – may explain so-called "viral autoimmunity", some classical autoimmune disorders and other diseases, including type II diabetes mellitus, and some forms of obesity. Viral receptor disease is a unifying hypothesis that may also explain some diseases with well-established, but multi-factorial and apparently unrelated aetiologies – like coronary artery and other vascular diseases – in addition to diseases like schizophrenia that are poorly understood and lack plausible, coherent, pathogenic explanations.

## Introduction

### 1.1 Global impact of RNA polymerases

Many of the world's population suffer from acute and chronic viral infection. The two common types of chronic viral hepatitis (CVH), hepatitis B (HBV) and C (HCV) are major causes of death and morbidity; conservative estimates suggest 400 million people are persistently infected with HBV, while HCV may infect a further 200 million. Annually, in excess of two million people will die from cirrhosis or liver cancer caused by CVH, and many more suffer chronic ill health as result. During the 20 years since the human immunodeficiency virus (HIV) was identified, perhaps 40 million people have become infected worldwide and each year about a million die from resulting immunodeficiency and consequent opportunistic infections, particularly tuberculosis, and other complications. Poor countries bear a disproportionate burden of disease caused by these viruses that further exacerbate poverty through pervasive economic disruption and diversion of limited resources to healthcare and disease control. Emerging viral pathogens including West Nile virus (WNV), the SARS coronavirus, endemic viruses like Murray Valley, Japanese, and other encephalitis viruses, Dengue and yellow fever, and seasonal influenza, hepatitis A (HAV) and E (HEV) cause enormous further morbidity and mortality, while pandemic outbreaks of virulent influenza strains remain a constant threat. Together, these viruses probably kill more people every ten days than the Boxing Day Tsunami. RNA viral infections, including Foot and Mouth, Bovine Viral Diarrhea Virus (BVDV) and Hog Cholera Virus (HChV), cause similar devastation of animal populations with enormous economic consequences.

RNA polymerases generate massive genetic variability of RNA viruses and retroviruses that circulate within infected hosts as vast populations of closely related, but genetically distinct, molecules known as quasispecies. After translation, this genetic variability causes near-infinite antigenic heterogeneity, facilitating viral evasion of host defences. Tuberculosis, malaria and other cellular pathogens also express broad cell-surface antigenic heterogeneity, generated by DNA-dependent RNA_pol_. Thus, RNA polymerases probably cause more morbidity and premature mortality in man, and other animals, and greater economic loss, than any other molecule.

### 1.2 RNA viruses and immune control

Despite a depressing global epidemiology that strongly suggests otherwise, the immune system is thought to "control" viruses. What practical meaning does "immune control" have for the individual? There is no argument for HBV, and other viruses, high affinity antibody, generated by prior vaccination or other exposures and directed against neutralizing epitopes, will prevent HBV infection (excepting vaccine escape mutations [1,2]), in part by blocking viral ligand  interaction with cell receptors, or that most patients exposed to HBV develop neutralizing antibodies (HBsAb), clear HBsAg from serum, and will normalize liver function long term. However, even patients who develop robust immune responses to HBV, defined by high-affinity antiHBsAb and specific antiviral cytotoxic T cell (CTL) responses, will have both "traces of HBV [3] ... many years after recovery from acute hepatitis" [3] and transcriptionally active HBV demonstrable in peripheral blood mononuclear cells (PBMCs) [4]. Furthermore, occult HBV is detected in liver tissue of patients with isolated antiHBc (i.e. HBsAg/HBsAb negative) [5] and in patients with HBsAg-negative hepatocellular carcinoma [6] suggesting, at least some patients, HBV in may persist irrespective of any immune responses, implying long term latency and low level basal replication may be a survival/reproductive strategy for HBV.

For most patients, acute HCV or HIV infection results in life-long viral persistence. Although many patients develop immunological responses, including specific antibody and CTL reactivity to various viral antigens, these responses have little discernible impact on either HCV or HIV replication that occurs essentially unchecked at rates estimated between 10^10 ^and 10^12 ^virions per day [7,8], indefinitely, while progressive destruction of liver or immune cells proceeds, commonly resulting in cirrhosis or liver cancer (for HCV) or death from immune deficiency (for HIV). Evidence that prior HCV infection confers no protective immunity against heterologous HCV infection in humans [9] or chimpanzees [10] or against either homotypic [11] or heterotypic [12] human reinfection, confirmation that active HCV infection persists long after either apparent spontaneous [13] or treatment-induced [14] viral clearance, or that vaccines causing specific antiviral B and T cell responses fail to protect against infection in animals [15], and that antibodies to HCV envelope protein E2 are only detected in animals with persistent infection [16,17], further undermines the potency of "immune control" and suggests, at least for patients with HCV, the definition of "control" may need to broadened significantly.

Based on observations that stronger specific CD4/CD8 immune responses with T-helper (TH1) cytokine profiles are found more frequently in patients with self limiting viral infections than those who develop chronic viral carriage [18,19] it is thought ability to mount robust adaptive immune responses predicts viral clearance while failure to do so results in chronic viral carriage [20]. However, detailed and very painstaking studies, albeit in small numbers of chimpanzees [21] and patients following antiviral therapy [22], have failed to demonstrate any relationship between T cell responses and viral clearance. Although development of TH1and other immune responses are certainly temporally and, probably, causally related to reduced viral replication and viral clearance the assumed direction of causality (immune response -> reduced viral replication), is not proved by the fact those responses develop, post hoc ergo propter hoc, as comforting a conclusion as it may be to reach.

The first part of this paper explores the impact of RNA_pol _fidelity on quasispecies behaviour, specifically in mediating immune avoidance during acute HCV infection. We suggest the primary event causing reduction in viral replication is inhibition of RNA_pol _processivity by variant viral proteins, specifically envelope and envelope-related proteins. We also suggest that immune responses to viruses are thwarted initially by broad antigenic diversity generated by low RNA_pol _fidelity but develop, when they do, after viral replication falls (because of reduced RNA_pol _processivity) and polymerase fidelity increases – linked events that occur because of replicative homeostasis – thus restricting antigenic diversity sufficiently to permit focused immune recognition. We further suggest immune responses strategically exploit replicative homeostasis to force viruses to reveal critical dominant antigenic epitopes, facilitating progressively more focused immune responses. The second part explores the ineluctable consequence of viral RNA quasispecies: That is, translation of RNAs into protein quasispecies with a spectrum of phenotypes and unpredictable properties, among which may be disruption of the cell surface receptors that viruses co-opt for cell entry. This innate property of viral quasispecies may explain a wide variety of diseases apart from viral autoimmunity.

## 2. Immunological, viral and biochemical kinetics following acute viral hepatitis

**Figure 1:**
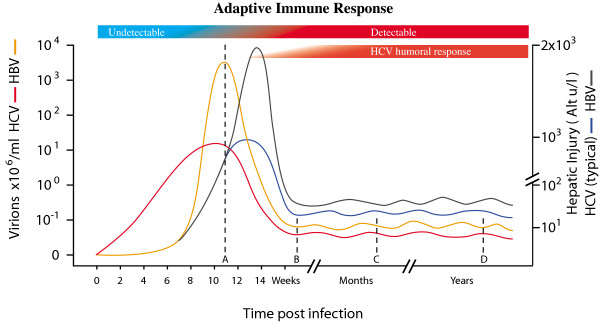
Viral replication, immunological and tissue injury kinetics following acute HCV and HBV infection. Data summated from Figure 1 [29] and modified to represent typical patients with chronic viral persistence. Note: a) High level HCV replication for 6–8 weeks prior to any immune responses, b) onset of humoral immune response well after down-regulation of viral replication [34], and c) transaminase peaks occurs ~ 2weeks later.

Acute HCV and HBV infection have characteristic kinetics of viral replication, adaptive immune responses, and cause predictable tissue injury, reflected in elevated serum aminotransferases. These kinetic and transaminase responses are summarized schematically for patients with persistent infection (figure 1) [23]. Initial HCV replication is very rapid and viral load increases exponentially until about week 4, at which point viraemia increases more slowly, and asymptotically, towards ~10^7 ^genome equivalents (geq)/ml by weeks 7–8 (these kinetics alone suggesting competitive inhibition of RNA_pol_). This exponential increase of viral RNA in serum reflects explosive dissemination of virus in tissues, detectable by in-situ hybridisation throughout hepatocytes, including the nuclei, within days of infection [24]. Viral replication declines rapidly from weeks 10–11 to weeks 14–16 falling by 10^2–3 ^geq/ml but lower level (~10^5 ^geq/ml) fluctuating replication persists, generally indefinitely, thereafter. By contrast, neither HBV DNA nor HBV antigens are detectable in either serum or liver for 4–7 weeks post infection [25,26]. Elevation of alanine aminotransferase (ALT), reflecting hepatocyte injury, is typically much greater for HBV than HCV, peaks about two weeks after replication of either virus declines. Fluctuating transaminase elevation – mirroring fluctuating viraemia in HCV infection [27] – often persists indefinitely. This kinetic profile contains three paradoxes:

### 2.1 The replicative kinetic paradox

**Figure 2:**
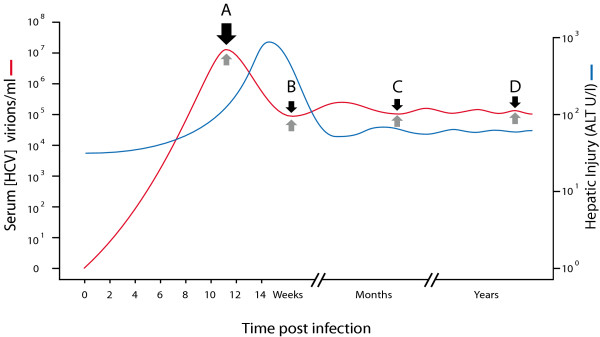
Paradoxical HCV replication kinetics. If host immune clearance forces (I_c_, black arrows) reduce viral replication acutely (point A), then they must exceed viral expansive forces (V_e_, grey arrows) at that point. At equilibrium (e.g. points B through D), viral concentrations (—) and, therefore, viral forces, have fallen by 10^2–3 ^hence, immune forces I_c _must fall by >10^2–3 ^from A to B for equilibrium to develop. There is no evidence this happens.

This has been described in detail previously, and relates to the replicative kinetics of HCV, HIV and HBV [28] and other viruses causing persistent infection. Briefly, and specifically for HCV, if immune functions are responsible for falling viral replication seen between point A to point B (figure 2), then the immunological clearance forces at point A must exceed the viral expansive forces (proposition 1). At points B to D (or any point between), where equilibrium develops, immune and viral forces must be equal, by definition (proposition 2). As viral concentration and, therefore, viral forces fall between points A and B to D by 10^2–3 ^geq/ml (observation 1), the immune forces must also fall by >10^2–3 ^between A and B to D for equilibrium to develop (proposition 3). There is no evidence this occurs, and very considerable evidence that immune force(s), as judged by development of specific cytotoxic T cell and antibody responses, are increasing during this time [29] (observation 2, proposition 4). Antecedent propositions (1–3) and (observation 2, proposition 4) are self-contradictory and incompatible with the conclusive belief that immune responses cause HCV replication to fall, hence either (a); the well-documented and multiply repeated observations of viral kinetics and adaptive immune responses are incorrect or (b); falling HCV replication beginning week 10 is not caused by host factors. Simply put, if immune or other host defences are able to clear virus at point A, why should they falter at B when less then 1% of initial viral load and antigenic diversity remain?

### 2.2 Temporal tissue injury (aminotransferase) paradox

Both HBV and HCV are non-cytolytic and viral clearance from hepatocytes, as well as hepatocyte injury, thought to be immune mediated. However, for both HBV and HCV the brisk fall in viral replication following acute infection precedes the peak of transaminase rise by at least two weeks (figure 1). If falling viral replication is due to adaptive immune responses causing hepatocyte lysis the transaminase peak should either precede or be coincident with falling replication. This temporal relationship is also inconsistent with the belief immune factors cause the falling replication seen during acute HCV or HBV, and is analagous to non-cytolytic reductions of viral replication observed for both HBV and lymphocytic choriomeningitis virus (LCV) experimentally, that suggested either [unspecified] antiviral mechanisms are operative [30,31], or that auto-inhibition of RNA_pol _by viral mechanisms (replicative homeostasis) occurs [28]. However, if other non-cytopathic host anti-viral mechanism(s) are responsible, the kinetic paradox implies their potency falls significantly between points A and B.

### 2.3 The Hepatitis C "early replication" paradox

Hepatitis C replication kinetics and their relationship to immune responses are well documented [32,33] but reveal an unexplained paradox. Despite high level viral replication, adaptive cellular immune responses to HCV are completely undetectable for at least 7–10 weeks [33] after infection, while humoral responses are rarely detected before 12–14 weeks [34], and in some patients [35], and some chimpanzees [36], are never detected at all. An exhaustive and very careful review of the clinical and experimental data relating adaptive immune response and HCV replication kinetics has been published recently [29]. Seeking to rationalize the enigma posed by a complete lack of immune responses to HCV replication of ~10^6–7 ^geq/ml at week 6 but [variable] immune responses to replication at ~10^5 ^geq/ml after week 14, the authors conclude "..[the data]...appear[s] to be consistent with the interpretation that HBV and HCV are ignored by the adaptive immune system for about 2 months after primary infection" and "[in HCV].. the adaptive response seems to really ignore for several weeks a substantial quantity of virus (at least 10^6 ^copies/ml)..". This is certainly an accurate synthesis of an extensive and highly complex literature but does it make any sense?

If adaptive immune responses really ignore high level HCV replication for two months, as suggested, then the following mechanism(s) are implied: a) an accurate mechanism for prompt detection of infection; b) A timing mechanism; c) A trigger mechanism for immune responses independent of any viral factor (given levels of virus are greater before immune recognition than afterwards the trigger for immune response must be either non-viral or falling (!) viraemia); and, as cytomegalovirus (CMV)-specific CD4(+) T cell responses arise within 7 days of CMV infection [37]; d) A mechanism allowing the immune system to differentiate HCV from CMV and other viruses (and reasons to do so). While possible, this seems unusually inelegant and pointlessly counterproductive, especially as events soon after infection probably determine whether virus is cleared or chronic infection develops. It is much more likely that adaptive cellular or humoral immune responses do not develop in the first 6–7 weeks of HCV infection simply because the virus isn't "seen". Why should HCV replicating at 10^6–7 ^geq/ml at week 6 be invisible to the immune system but visible when replicating at 10^5 ^geq/ml long term? Dissection of this problem requires explicit analysis of what is being measured and how.

### 3.1 Hepatitis C: measurement and detection

Assay of HCV RNA and detection of HCV by immune responses measure two quite different things. Quantitation of HCV is typically performed by branch-chain cDNA assay (bDNA) or quantitative PCR (qPCR) using probes or primers complementary to conserved 5'untranslated (5'UTR) HCV RNA sequences. Immune responses to HCV typically "measures" envelope proteins translated from envelope-encoding RNA (EeRNA) sequences and are directed at specific antigenic amino acid sequences and polypeptide conformations, not total viral envelope protein concentrations. While concentrations of 5'UTR RNA will be proportional to EeRNA concentrations in any given sample, they may not be identical for two reasons; i) RNA transcription may prematurely terminate making 5'UTR RNAs relatively more prevalent than EeRNAs and ii) HCV 5'UTR is highly conserved, while EeRNA s are less constrained, making hybridization efficiencies of PCR primers or bDNA probes greater for 5'UTR RNAs than for the population of EeRNAs, causing relative under-estimation of true envelope RNA concentration^1^. Nonetheless, as 5'UTR HCV RNA concentrations will be proportional to EeRNA concentration, the question remains; why should envelope proteins translated from EeRNA sequences present at concentrations corresponding to ~10^5 ^5'UTR geq/ml at 16 weeks be visible immunologically, but envelope proteins derived from EeRNA sequences corresponding to ~10^6–7 ^5'UTR geq/ml at 4–6 weeks remain unseen? Quasispecies biology, specifically variable RNA_pol _fidelity, replicative homeostasis, and sequence-specific requirements for both genetic and immunological detection suggest an answer.

## 4.0 Quasispecies biology: Generation of genomic and phenotypic diversity

**Figure 3:**
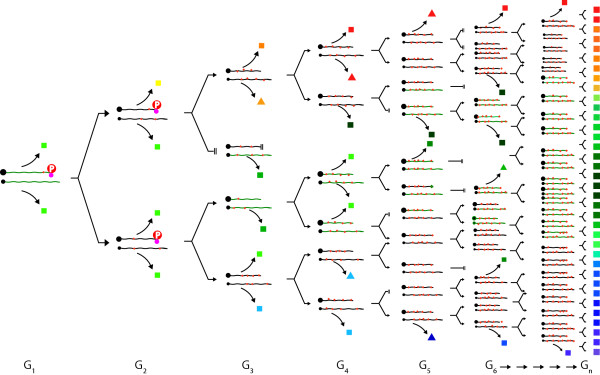
Simplified, two dimensional clade diagram of hyperdimensional viral RNA protein sequence-space. Because of RNA_pol _(P) infidelity and Müller's ratchet, mutations () are introduced into each RNA template synthesized, and progressively accumulate, resulting in an RNA quasispecies with sequence progressively divergent from consensus sequence. Translation results in a spectrum of proteins (, , , etc.) with properties that also vary progressively from wild-type sequence () to highly variant proteins (, , etc.). Some RNAs will be so abnormal that translation or replication fails or is truncated (), while others will code for grossly defective proteins ( ,  etc.).

RNA viruses replicate by copying antigenomic templates, a process catalysed by RNA_pol_, an enzyme lacking fidelity or proof reading function [38-41]. Theoretically, an RNA viral genome like HCV (about 9200 bases) could assume any of 4^9200 ^(about 8.95 × 10^5538^) possible sequence combinations exceeding, by some margin, population estimates of protons in the known universe (about 10^80^), meaning the potential complexity of RNA viral quasispecies is infinite, for all practical purposes. An RNA_pol _fidelity rate of 10^-5 ^errors per base copied predicts at least one and as many as 10 (estimated for HIV) [39] genomic mutations will be introduced during each cycle of replication. Furthermore, as HCV replication results in synthesis of ~10^12 ^virions per person per day [8], on average, mutations will develop at each genomic locus ~10^7 ^times/day, while the probability any two genomes synthesized consecutively will be identical is about 10^-6^. The sum effect is inexorable accumulation of genomic mutations – that, by itself, should threaten replicative fitness because of Muller's ratchet [42] – and progressive dilution of wild-type genomes (figure 3), processes that make long-term stability of RNA virus quasispecies highly paradoxical [43]. As argued previously, a combination of selective genomic replication and variable RNA_pol _fidelity, both mediated by replicative homeostasis, act together to prevent RNA quasispecies extinction [28].

The phenotypic consequences of viral quasispecies biology may be more important. Progressive divergence of genomic RNA sequences away from wild-type sequences caused by RNA_pol _infidelity generates a massive population of closely related, but genetically distinct, RNA molecules (figure 3), an effect operative at all scales from each open reading frame (ORF) to whole virus species. A quasispecies of ORF RNAs has but one inevitable outcome; translation of a quasispecies of viral proteins with a vast and highly variable spectrum of phenotypes, some subtly nuanced, others grossly defective. Furthermore, mutations that create new, or obliterate pre-existing, start or stop codons in a significant proportion of RNAs, will cause translation of highly unusual and heterogeneous proteins, particularly during high-level viral replication, a phenomenon that may explain HBeAg. Viral quasispecies cannot, and will not, produce homogeneous proteins with predictable and consistent phenotypic and antigenic properties.

### 4.1 Quasispecies biology: Frequency distribution of genomic and phenotypic diversity

**Figure 4:**
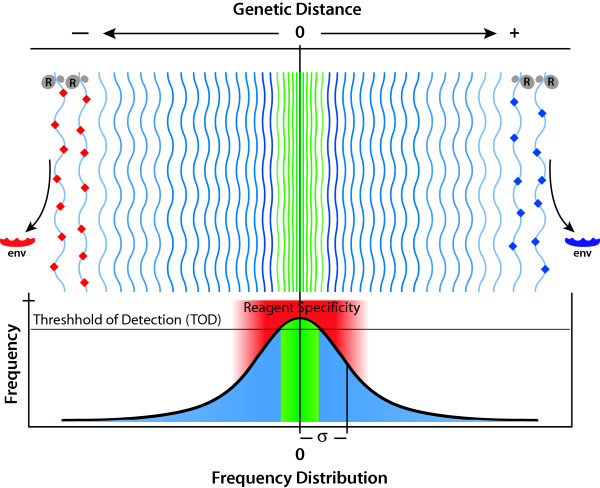
Two-dimensional representation of hyperdimensional RNA (or corresponding protein) frequency distribution curve (scale arbitrary) with conceptual centre of gravity of replication (wild type, green) and variant sequences (blue), zone of reagent specificity (red shading) and threshold of detection (TOD) of any assay. As mutations ( , ) accumulate and RNA sequence progressively diverges from consensus sequence (0) the probability of that RNA sequence and corresponding protein (e.g. envelope, Env.) arising falls rapidly. Standard deviation (σ) of frequency distribution is proportional to RNA_pol _fidelity.

While RNA_pol _infidelity will cause progressive divergence of copied sequences away from wild-type or consensus sequences, the probability of any particular sequence arising will fall dramatically with increasing genetic distance from that consensus sequence (figure 4), allowing conceptual representation of the resulting genomic (and consequent phenotypic) diversity as a frequency distribution curve, with increasingly variant sequences surrounding a 'centre of gravity of replication', formed by wild-type sequences. Viral quasispecies occupy hyperdimensional sequence-spaces, hence any physical representation is necessarily simplified, but because mutation away from wild-type sequences is equally probable in all directions, variant RNA and protein frequencies will be normally distributed and the standard deviation (SD, σ) – insofar as 'normal' or 'standard' can be applied to a hyperdimensional space – of that distribution will be a function of RNA_pol _fidelity; if RNA_pol _is completely faithful, the RNAs and proteins will be monoclonal and σ = 0; if RNA_pol _has no fidelity, RNA will be synthesised randomly, and all RNA and consequent protein sequences will arise with equally probability, therefore σ = ∞. While viral RNA and related protein sequences are theoretically unconstrained (at least before any consideration of functionality), the sequence specificities of any reagents used in their detection (bDNA probes, PCR primers, mAbs etc) are not, by definition, and their specificity and the efficiency with which they detect variant molecules will fall progressively the further those variant sequences are from the consensus sequence. A zone of 'reagent specificity' may therefore be defined probably encompassing wild type and some variant sequences, but there will exist some RNA sequences and corresponding proteins of any quasispecies that are undetectable with these sequence-specific reagents. A threshold of detection of any assay (including immune detection) may similarly be defined; RNA or protein sequences present at concentrations below this conceptual level being undetectable by that particular assay. The HCV "early replication" paradox now partially resolves; the 5'UTR sequences are both highly conserved and common to virtually all RNAs in the quasispecies, therefore, the 5'UTR concentration – that is, the common measure of HCV viraemia – corresponds to the area under the frequency distribution curve. By contrast, envelope RNA sequences (and related envelope proteins) are not so constrained and their relevant concentrations (i.e. whether or not that RNA or protein sequence is detectable) corresponds to the frequency of that specific sequence in the quasispecies and that, in turn, depends on RNA_pol _fidelity; if RNA_pol _fidelity is low, the frequency or concentration of any particular RNA or protein sequence will also be low and may be below the detection threshold, while increasing RNA_pol _fidelity may increase sequence frequency [i.e. the concentration of specific proteins] above detection threshold. But why should specific EeRNA sequence frequencies – in other words, HCV RNA_pol _fidelity – increase after week 8, facilitating adaptive immune responses? Viral autoregulation, specifically replicative homeostasis, provides an answer.

## 5.0 Co-evolutionary adaptation

Interactions among species, whether between humming birds and flowering plants, primitive viroids and prokaryotic cells or HCV and man, results in an unremitting process of adaptation and responsive counter-adaptation – in effect, a molecular arms race – for each species just to maintain ecological parity. The price of survival for a species is continual evolution. Survival, for viruses, requires cell entry, a precondition long antedating necessity to evade more complex host defenses, including interferons and other cytokines and adaptive immune responses, while for cells, and complex cellular organisms, cell wall defenses, including receptor polymorphisms, form a principal barrier against viral invasion. Viral survival – effectively meaning RNA_pol _survival – on an evolutionary timescale, as argued previously [28,44], requires control of mutation and replication rates in a manner adaptively responsive to constantly changing biota and this implies dynamic linkage of RNA_pol _fidelity and processivity with quasispecies phenotypic and antigenic diversity, meaning an autoregulatory linkage – Replicative homeostasis – between RNA_pol _fidelity and processivity and envelope proteins, as argued previously [28]. By definition, evolutionary co-adaptation occurs in response to adaptations in locally prevalent interacting species. Natural selection for beak variation(s) in Darwin's finches occurs as a consequence of concrete survival benefits these variations – mediating, for example, enhanced food harvesting interactions with other variable plant or animal species – confer to individual Galapagos Island birds, rather than any inexorable hypothetical 'improvement' in beak function for finches in general. If a species is widely distributed in space, but population mixing is slow or incomplete, locally prevalent interactions with other species will vary and regional genetic variations will arise and be maintained, hence progressive divergence from the original genotype (speciation) may result. For viruses, and their hosts, genetic variations – reflected in viral genotype and cell surface polymorphisms and resulting disease susceptibilities – would be predicted, and are observed [45-50], to have frequencies that vary geographically.

### 5.1 Enzymatic Autoregulation

**Figure 5:**
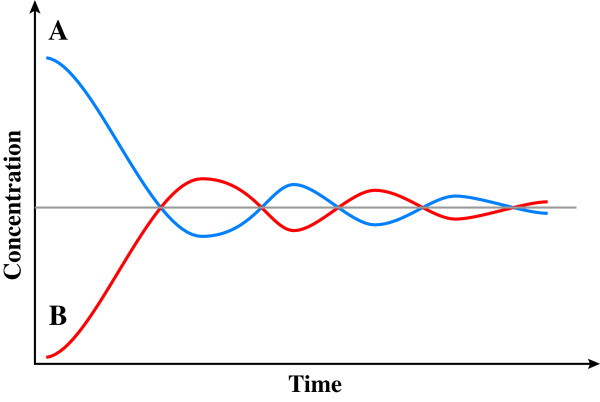
Autoregulation of a simple enzyme system: If enzyme E produces either A () or B () and product:enzyme interactions occur such that A:E produce B while B:E favour A, then high initial concentrations of A (or B) will cause rapid synthesis of B (or A). Equilibrium ultimately develops irrespective of starting conditions.

Consider the following; An enzyme (E) functioning in a closed system synthesizes either product A or B that both interact with E to influence output such that A:E interactions cause production of B, while B:E interactions produce A. Irrespective of starting conditions (excluding substrate exhaustion and product inhibition), an equilibrium will eventually develop (Figure 5) with the relative concentrations of A:B determined by the relative association constants (K) of A:E (K_A:E_) and B:E (K_A:B_) and the velocity (ν) of production of A from B:E (ν_A_) and B from A:E (ν_B_). Removal or addition of either A or B will alter equilibrium conditions but not the fact equilibrium is reached; if A is removed, for example, the increased frequency of B:E interactions will cause compensatory increased A synthesis; in this sense enzymatic autoregulation occurs. Intuitive analysis suggests that enzymes acting in a milieu of increasing concentrations of inhibitory molecules become progressively less processive until reduced enzyme output is insufficient to further inhibit enzyme activity, and an equilibrium state is reached. Considering viral replication, if alteration of RNA_pol _fidelity causes synthesis of either wild-type or variant RNA sequences (simplified, as a continuum between these two must exist) that are subsequently translated into either wild-type or variant polypeptides that then interact with RNA_pol _such that wild-type: RNA_pol _are high affinity interactions that induce rapid, low fidelity RNA_pol_ replication while variant protein: RNA_pol _interactions are low affinity and cause high fidelity RNA_pol_ replication at low rate then an equilibrium will eventually develop. Hence, as relative concentrations of wild-type and variant viral proteins vary, alteration of both processivity and fidelity of RNA_pol _results, permitting viruses to adaptively respond to environmental changes, including immune recognition and reaction to evolving cell receptors. Stable, highly reactive equilibria not only develop as a result of RNA_pol_/envelope interactions and viral autoregulation, there is no option but for this to occur.

### 5.2 Co-evolutionary adaptation: Cell-surface polymorphisms

Generation and maintenance of polymorphisms, that is, replacement of existing genes – that, by operational Darwinian definition, have proved their functionality and evolutionary fitness by surviving to reproduce – with variant genes (polymorphisms) of uncertain functionality, fitness or overall compatibility within an organism, is an evolutionary strategy that will only be sustained on a geological timescale if new polymorphisms confer survival benefits to organisms that exceeds the risks and metabolic costs of generating and sustaining those polymorphisms. For primitive cells, lacking functional humoral, cellular or cytokine defense mechanisms, development of cell-surface protein polymorphisms is an obvious adaptive strategy to thwart invasion by primitive viruses. Like other adaptive strategies, cell-surface polymorphisms are strongly selected for, and have been highly conserved over deep time, and are found in all organisms from primitive prokaryotic cells [51] and thermophilic bacteria [52] through to plants [53] as well as mammalian cells, strongly suggesting a critical evolutionary function. The lock and key hypothesis, for which there is very considerable evidence [54-57], first proposed by JBS Haldane [58], contends polymorphisms arise, and are maintained, as protection against cellular parasitism, particularly by viruses^2^. While DNA-encoded protein polymorphisms form necessary defenses against viral access, they may not be sufficient; a quasispecies of cells (e.g. the liver) expressing similar and static receptor variations renders those cells vulnerable to sustained attack from any virus that successfully invades any one cell, and further dynamic modification of cell receptors, triggered by viral infection and mediated at the transcriptional level by modulation of DNA dependent RNA polymerase fidelity in nearby uninfected cells, by a mechanism similar to replicative homeostasis would seem possible.

## 6.0 Problems of Detection

A clear, unambiguous band at the "C" position on a sequencing gel, causes "cytosine" to be assigned to that genetic locus. But does this certitude reflect reality, at least for viral RNA quasispecies? Direct PCR sequencing is an "averaging" procedure revealing the most frequent nucleotide at any particular locus. However, nucleic acids and proteins cannot express 'an average', and discrete quanta of specific nucleotides or amino acids are present at every locus. A typical clinical serum sample, containing 4 × 10^5 ^geq/ml HCV and mutating at 10^-5 ^substitutions/base, will contain examples of each possible nucleotide at every locus, but most variations will remain undetected during sequencing or any other method of quasispecies analysis. Analysis of cloned DNA gives cleaner data than PCR sequencing but if 100 clones (and multiple HCV quasispecies clones are highly unlikely to be identical) provides definitive sequence, would we process the 101^st ^to reveal different and, potentially, critical sequence variations? And if we did, how would we recognise its importance? Is important sequence likely to be present at frequencies of < 1%? Infectious virions containing, presumably, full-length functional genome and corresponding wild-type proteins, are often outnumbered by ~6 × 10^4^:1 in serum by defective and non-infectious particles [53] that presumably do not, suggesting that important genetic sequence and associated phenotype may occasionally be extremely rare. How the immune system recognizes uncommon, nondescript, but important protein sequences in a featureless background of similar molecules is a non-trivial problem for which replicative homeostasis may suggest a solution.

## 7.0 Replicative Homeostasis

**Figure 6:**
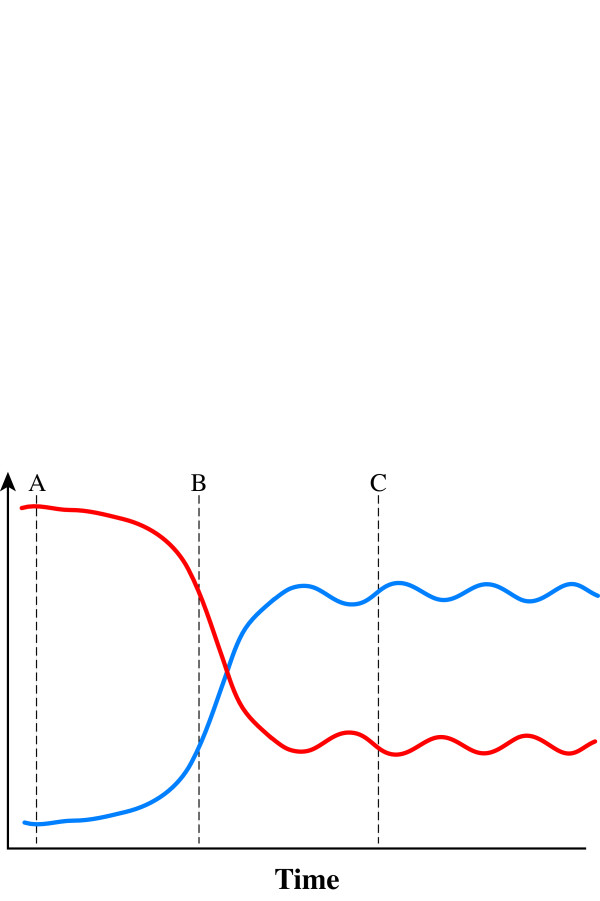
Dynamic progression of RNA_pol _functional properties, processivity () and fidelity () predicted by replicative homeostasis. Initial state (A, corresponding to panel A, Figure 7): in a newly infected cell, high-affinity wild-type:RNA_pol _interactions will predominate resulting in high RNA_pol _processivity but low fidelity causing high-level viraemia with broad virus phenotypic spectrum, maximizing cell tropism. Intracellular accumulation of variant viral proteins (B, c.f. panel B, Figure 7) reduces RNA_pol _processivity but increases fidelity reducing viral RNA synthesis and consequently, viraemia before a dynamic, fluctuating equilibrium (C, c.f. panel C or D, Figure 7) develops in which inhibition of RNA_pol _by variant viral proteins is balanced by increases in RNA_pol _fidelity (with consequent synthesis of wild-type viral products tending to cause high RNA_pol _processivity).

**Figure 7:**
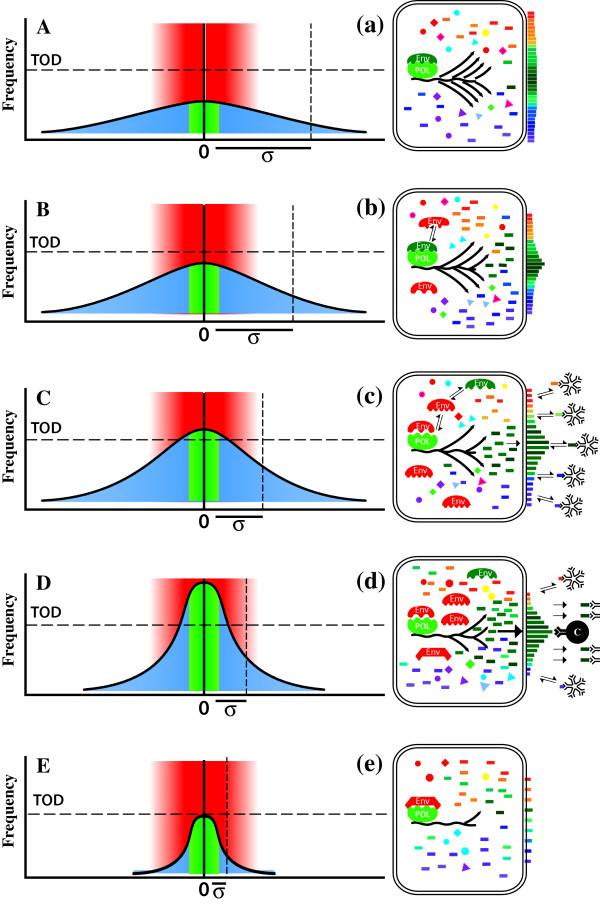
Conceptual progression of intracellular viral replication events, including variable RNA_pol _fidelity and processivity, restriction of antigenic diversity and immune recognition under influence of Replicative homeostasis. Panels (A->E) changing frequency distribution of viral RNA and protein quasispecies, panels (a->e) cellular events. Initial state (panels A,a) viral replication occurring in cells devoid of molecular inhibitors of RNA_pol _high affinity wild-type envelope (Enve, green): RNA_pol _interactions predominate, causing rapid low-fidelity viral RNA synthesis and, consequently, a broad spectrum of viral proteins expressed on cell surface at concentrations below TOD. As variant viral proteins accumulate within cells (panel b) and variant viral envelope: RNA_pol _interactions increase, RNA_pol _fidelity increases while processivity decreases, restricting the distribution of viral RNA and proteins, reducing antigenic display on cells. As variant viral envelope: RNA_pol _predominate (panel c), the frequency distribution of expressed viral proteins is restricted so the individual concentration of some proteins increases beyond TOD, allowing immune recognition and polyclonal, low affinity antibodies to develop, blocking cellular egress of viral proteins, further increasing variant viral envelope: RNA_pol _interactions, thus immune responses force viruses to reveal wild-type epitopes by restricting antigenic diversity. High affinity responses once developed (panel d) preferentially reduce intracellular concentration of wild-type viral proteins further increasing variant viral envelope: RNA_pol _interactions still further restricting RNA_pol _processivity to the point of viral latency (panel e).

Replicative homeostasis, described in detail elsewhere [28,44], is an epicyclic mechanism of viral autoregulation that results when viral proteins, notably envelope (Env), influence RNA_pol _fidelity and processivity. The predicted consequences of replicative homeostasis for rates of intracellular viral replication and mutation, cellular expression of viral proteins and immunological responses occurring because of replicative homeostasis is represented schematically (figures 6, 7). During early viral replication in a naive cell devoid of inhibitory molecules (panel A, a), high affinity wild- type envelope:polymerase interactions predominate, causing rapid low-fidelity polymerase activity resulting in rapid synthesis of variant viral RNAs and subsequently proteins, hence causing a broad spectrum of viral proteins to be expressed on the cell surface, each at concentrations below the threshold of immune detection (TOD). RNA_pol _infidelity ensures synthesis of variant viral RNAs and proteins predominates early, hence variant protein molecules progressively accumulate within cells relative to wild-type viral molecules (Panels B-D) and increasing the probability of variant viral envelope:RNA_pol _interactions. Variant viral envelope:RNA_pol _interactions causing progressive inhibition of RNA polymerase processivity and increasing RNA_pol _fidelity, reducing diversity of viral RNAs synthesized and progressively restricting viral protein diversity expressed on the cell surface (panels b to d), increasing cell-surface concentrations of individual viral proteins above the threshold of detection (panels C, c) at which point a polyclonal immune response develops. Development of low-affinity polyclonal blocking antibodies, restricting cellular egress of viral proteins, further increasing intracellular concentrations of variant envelope proteins, still further increasing the probability of variant viral envelope:RNA_pol _interactions and inexorably further restricting antigenic diversity increasing relative expression of wild-type proteins thus further exposing these epitopes to immune surveillance and facilitating specific high-affinity immune responses, including cytotoxic T cell responses, (D,d) to wild-type proteins. Thus, the immune responses can strategically utilize replicative homeostasis to force viruses to reveal important and dominant wild-type epitopes, but those responses develop initially as a consequence of restriction of RNA_pol _fidelity that occur because of replicative homeostasis. High-affinity responses further deplete intracellular concentrations of wild-type proteins, progressively reducing wild-type envelope:RNA_pol _interactions, greatly reducing RNA_pol _processivity to the point of viral latency (E,e), caused by variant viral envelope:RNA_pol _interactions.

## 8.0 Discussion

The hepatitis C "early replication" paradox now resolves completely when considered in the context of replicative homeostasis; initial high level HCV replication (due to high RNA_pol _processivity) remains immunologically undetectable for 6–8 weeks, or more, because of low RNA_pol _fidelity causing a broad spectrum of HCV envelope proteins each expressed on cell surfaces at concentrations below the threshold of detection even while viraemia, reflected in concentrations of 5'UTR RNA common to each RNA species, are present at 10^6–7 ^geq/ml. As replication progresses, intracellular accumulation of variant viral proteins increase RNA_pol _fidelity but decrease processivity (replicative homeostasis), downregulating HCV replication and reducing viraemia but restricting antigenic diversity and increasing expression of HCV envelope proteins to beyond the threshold of immune detection. Furthermore, the temporal tissue injury (aminotransferase) paradox also resolves in this light: Focussed immune recognition (including cytotoxic T cell responses) doesn't develop until after viral antigenic diversity is restricted by replictive homeostasis the transaminase peak would not be expected until after viral replication falls due to autoinhibition of RNA_pol _processivity. Varying expression of viral proteins by modulating RNA_pol _fidelity to facilitate immune escape would seem a useful evolutionary adaptation that might be retained by more complex organisms, including cellular pathogens like tuberculosis and malaria, to optimize their stability within hosts.

This mechanism of immune avoidance might also explain maternal-foetal tolerance. The human foetus maintains a stable parasitic existence during gestation (and, I expect, to University age and beyond) that is tolerated despite normal maternal immune responsiveness in general and lack of specific tolerance to paternal antigens in particular, a situation made more problematic as expressed foetal antigens are predominantly of paternal origin [54]. While immunological isolation of foetal tissue by the placental trophoblastic layer [55], and placental display of HLA-G [56], probably contribute to foetal stability in the face of a potentially robust immune attack, neither mechanism would explain persistence of viable foetal nucleated red blood cells within the maternal circulation [57] in quantities sufficient to permit clinical prenatal diagnosis [58]. Is it possible foetal tolerance is mediated by regulating the fidelity of foetal DNA dependent RNA transcriptases to ensure any cell-surface antigens are expressed heterogeneously and at levels below the threshold of maternal immune responsiveness?

## 9.0 Autoimmunity

For many classical autoimmune disorders, including primary biliary cirrhosis [59], multiple sclerosis, and rheumatoid arthritis, convincing epidemiological evidence [60], including cases clustering [61,62], strongly suggests these diseases are triggered by infectious agents in genetically predisposed individuals. In others, such as diabetes mellitus, tantalizing epidemiological [63], clinical [64] and laboratory [65] evidence has implicated enteroviruses, but has suggested viral-triggered autoimmune processes, rather than cytolytic destruction of pancreatic beta-cells [66]. Similar circumstantial evidence exists for myocarditis, demyelinating diseases, myositis and other post infectious inflammatory disorders. When MacFarlane Burnet wrote autoimmunity arises from "inability to distinguish self from non-self" HBV, HCV, HIV and other viruses, now established to cause diseases with clear autoimmune features were unknown. Viral infections, particularly hepatitis C – and its treatment with interferon – are associated with many varied autoimmune phenomena [67], and thyroid disease [68-70], diabetes mellitus [71,72], membranous, membranoproliferative and cryoglobulinemic glomerulonephritis, vasculitis and peripheral neuropathy [73], and autoimmune gastritis [74] are all very well documented, although the mechanism(s) are unknown and causality is certain. Classical serological markers of autoimmunity, including rheumatoid factor, antinuclear antibodies (ANA), anticardiolipin, antithyroid, anti-liver/kidney/microsomal antibodies (anti-LKM), as well as HCV/anti-HCV immune complex formation and mixed essential cryoglobulinemia are common accompaniments of chronic HCV infection [73], raising the obvious question of whether all "autoimmunity" has a viral basis. Indeed, Zinkernagel's pragmatic and subtly anticipatory; "If we know the infection, we call the disease immunopathologically mediated; if we do not recognize or know it, we call the disease autoimmune [75]" fully reflects recent explosive growth of information and the deeper questions this information poses.

## 10.0 Virus receptor disease

RNA virus quasispecies biology, specifically the generation of RNA quasispecies by RNA_pol_, and translation of these immensely variable RNAs into protein quasispecies, suggests an immediate solution to the problem of viral autoimmunity and, by extension, to autoimmunity in general, as well as suggesting a unifying hypothesis to explain other diseases known to have multi-factorial aetiologies that include inflammatory components – such as coronary artery disease – in addition to other diseases – including schizophrenia and some forms of depression – that currently lack rational and coherent pathogenic explanations.

Viruses are known to co-opt cell surface molecules, including lectins, hormone receptors and cell signaling molecules, to access cells. Receptors, and other cell surface molecules, identified as "viral receptors"or to specifically interact with viral proteins include prostaglandins, catecholamines and acetylcholine receptors [76], serotonergic neurotransmitters (5HT) [77], endothelial cell glycoproteins [78], insulin-like growth factor (IGF-IR) and its major signaling molecules insulin receptor substrates IRS-1 [79] and IRS-2 [80], epidermal growth factor (EGF) [81], neurotrophin receptor [82], thyroid hormone receptor TRalpha1 [83], an immunoglobulin protein superfamily [84], low density lipoprotein (LDL) receptors [85,86], transferrin receptor (TfR) [87], asialoglycoprotein receptor (ASGP-R) [88,89], and angiotensin-converting enzyme 2 [90], to cite biologically diverse examples. Of necessity, some receptor affinity studies have used cloned viral protein ligands, an artificial situation that cannot approach the phenotypic complexity of RNA viral protein quasispecies. Nonetheless, variable virus receptor affinities [91,92], evolutionary adaptation of receptor affinity [93], emergence of escape variants with altered receptor affinities [94], temporal alteration of receptor usage [92] and capacity to exploit alternative entry pathways [95] have all been confirmed, suggesting viruses are capable of generating highly plastic ligands with very broad receptor affinities.

If a virus co-opts a receptor for cell entry, then wild-type envelope (consensus sequence) epitopes, coded for by wild-type RNA sequences, will probably form the common viral ligand. However, any viral RNA quasispecies also contain a vast spectrum of RNAs derived from, and similar to, envelope open reading frame (ORF) consensus sequence, but variant from it. As the envelope ORF quasispecies sequences progressively diverge from wild-type, the quasispecies of envelope proteins translated from these variant ORFs will also, and inexorably, diverge in sequence, structure and biological function from wild-type envelope sequence proteins. Some of these envelope proteins will be functionally identical, but others, and probably the vast majority, will range from subtly different to grossly abnormal, either due to major differences of sequence and/or chemical or steric amino acid incompatibility, or because of premature introduction of stop codons. Even minor amino acid differences, as sickle cell anaemia illustrates, and has been confirmed specifically for viral receptor usage [96,97], may catastrophically alter a proteins' function with respect to co-opted viral receptors, with some having no binding affinity, while others will bind strongly and act as agonists, antagonists or competitive inhibitors of normal receptor function. Variant and defective viruses, and their polypeptides, will be in vast molar excess compared to wild-type [53] but will exhibit similarly high antigenic variability, permitting escape from immune and other scavenger mechanisms. As many variant viral polypeptides will bind tightly to "self" receptors, but contain immunogenic non-self motifs, a polymorphic (because variant viral proteins will themselves be highly polymorphic due to the quasispecies process) immune response, apparently directed against "self" antigens, but actually targeting virus protein-receptor complexes virtually indistinguishable from normal cell receptors, will result causing apparent 'autoimmune' tissue damage.

This mechanism suggests an explanation for common autoimmune phenomena. If a virus enters cells because wild-type envelope motifs interact with insulin, insulin receptor substrate [79,80], TSH or related molecules [83], or acetylcholine [76] receptors, many variant envelope polypeptides, generated by envelope ORF quasispecies RNAs, would have similar receptor binding affinity, but may effectively disrupt receptor function, predictably causing impaired glucose tolerance or diabetes mellitus, thyroid dysfunction, or myasthenia gravis with secondary resistance to, and elevation of, the normal hormone ligand (insulin, TSH etc.). The expected consequences disruption of receptor function by variant viral proteins might explain many common biochemical pathologies; For example, what effect would chronic blockade of parathyroid (PTH) receptors by viral proteins have on PTH levels, the parathyroid glands, or bone?

Leptin is a 16Kda protein hormone secreted by adipocytes and carried across the blood-brain barrier by a rate-limiting transporter to act on hypothalamic receptors [98] where, among other functions, it regulates thyrotropin-releasing hormone (trh) genes and upregulates alpha-melanocyte-stimulating hormone and other anorexigenic neuropeptides [99] important to appetite-regulation and energy balance [100]. Leptin also regulates a broad spectrum of other processes and behaviours including thermogenesis, blood pressure and immune function. s=Serum leptin concentrations and leptin resistance, are independent markers of obesity, weight gain, systemic hypertension [101], diabetes mellitus [102], obstructive sleep apnoea [103] and myocardial infarction [104], while polymorphisms of the leptin gene are associated with insulin resistance [105] and long-term risk of developing diabetes mellitus [102]. Predictably, variant envelope proteins generated by envelope ORF RNA quasispecies from viruses utilizing leptin receptors for cell access would have similar receptor affinity, but exhibit non-physiological leptin antagonist or agonist properties, thus disrupting leptin receptor function, altering energy regulation, and causing either excess caloric intake unrestrained by satiety responses, or inappropriate satiety signals with pathologically reduced caloric intake. As clear evidence exists for viral disruption of leptin function [106] and virus-associated weight gain in humans [107] and monkeys [108], is it possible the global epidemics of type II diabetes mellitus, insulin resistance, hyperlipidaemia and obesity now prevalent [109-116], are just that; epidemics fundamentally caused by viruses that co-opt insulin or leptin or other associated receptors for cell access and generate protein quasispecies that disrupt receptor function? Could it also be that ethnically based epidemics of obesity, diabetes mellitus, hypertension and reno-vascular disease (the 'metabolic syndrome'), as seen in PIMA Indians, Nauruans and Australian Aborigines [115] have developed not primarily because of exposure to "Western" foods and lifestyles – that, after all, are all-pervasive without necessarily having so dramatic an effect on other groups – but because of chronic or recurrent exposure to viruses, or genotypes of viruses to which their particular repertoire of receptor polymorphisms confer no protection? Or that anorexia nervosa develops, in some patients, when variant viral proteins with aberrant leptin-agonist function arise during the course of viral infection, as the temporal relationship between infection and disease onset, very clearly documented in one study [117], suggests.

Cardiovascular disease, the leading cause of premature death and disability in most western countries, has a well-established multi-factorial basis involving a complex interplay between genetic predisposition, environmental and personal risk factors – including systemic hypertension, diabetes mellitus, hyperlipidaemia, obesity and cigarette smoking – and more recently recognized mechanisms, including endothelial dysfunction [118], vascular inflammation [119] and leptin levels [104]. Systemic hypertension, diabetes mellitus and hyperlipidaemia have long-established, but complex, patterns of inheritance, a situation further compounded by evidence receptor polymorphisms – including those of angiotensin II type 1 receptor [120], IRS-1 gene [121] and low density lipoprotein receptor (LDLR) [122] – both confer disease susceptibility and have regionally variable prevalences [123,124].

**Figure 8:**
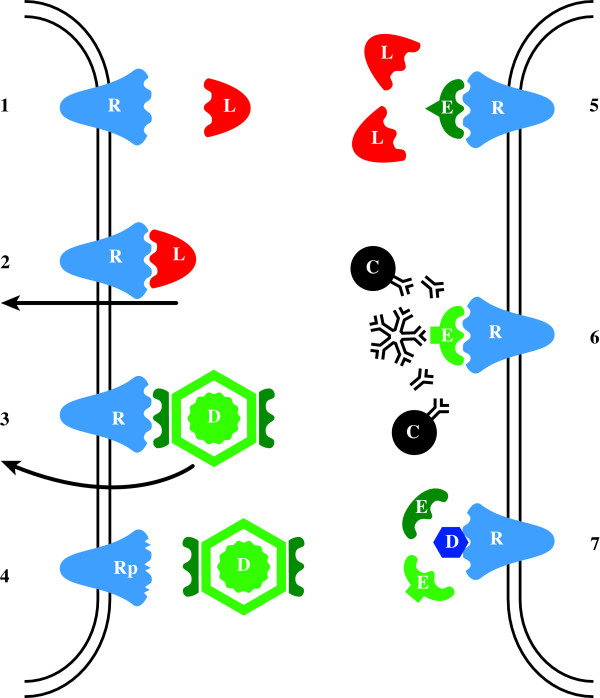
Cell receptor (R) and normal ligand (L; insulin, PTH, leptin etc.) relationship (1; unbound, 2; activated), receptor permissive for virus cell entry (3) or blocked by polymorphism (Rp, 4). Receptor blockade by variant viral envelope proteins (green E, 5), blockade by antigenic envelope proteins stimulating "autoimmune"response apparently directed against self receptors (E, 6), competitive displacement of antigenic proteins by drug (D, e.g. statin, aspirin) abrogating immune response (7).

The flaviviradae – including HCV – as a family, and the rous sarcoma virus, utilize low density lipoprotein receptors to enter cells [85,125], while angiotensin II [90], insulin receptor substrates (IRS1 and IRS 2) [79], and endothelial cell glycoproteins [78] and other receptors widely distributed in vascular tissues are known to be permissive for virus cell entry establishing, in principle and in fact, viral-protein receptor affinity relevant to cardiovascular diseases. Viruses accessing cells through these receptors will generate a quasispecies of variant proteins capable of disrupting receptor function potentially causing hyperlipidaemia, hypertension, hyperglycaemia and endothelial dysfunction, as well as immune-mediated endothelial cell damage, thus establishing the necessary and sufficient conditions and a chain of events that potentially link viruses and vascular diseases, including myocardial infarction. This hypothesis exists at the confluence of established risk factors for coronary artery disease, including genetic susceptibility, polymorphisms predisposing to hypertension [126-128], diabetes [126] and hypercholesterolaemia and substantial new data implicating vascular inflammation [119,129], endothelial dysfunction [119,130], leptin dysregulation [104] and viral infection [131,132] in the pathogenesis of vascular disease. Furthermore, this final common pathway can account for that small, but significant, group of patients with vascular diseases but no clinically identifiable risk factors, as well as the non-random co-incidence of depression and coronary artery disease [133] (as discussed below) in addition to the anti-inflammatory action of HMG-CoA reductase inhibitors (statins) [134], and their effect in lowering cardiovascular mortality independent of cholesterol reduction [135]; if statins compete with variant viral proteins for HMG-CoA reductase receptor binding, and displace immunologically attractive molecules, inflammatory responses directed at viral product, but involving endothelial cell receptors, will be ameliorated (figure 8).

Human immunodeficiency virus HIV-associated dementia (HIVD) occurs in 15% of HIV-infected adult patients, and as a major cause of dementia in the young represents "proof of principle" of virus-caused dementia, raising the possibility other forms of virus related dementia exist. Although highly active antiretroviral therapy (HAART) has reduced the incidence of HIV-D by 40–50% [136], it remains a major cause of morbidity and the pathogenesis poorly understood. Direct cytopathic effects of HIV or other viruses are unlikely, while active replication of virus, high-level viral protein expression [137], and increased viral envelope sequence-diversity in blood and brain [138] are all important, clearly indicating viral proteins are pathogenically important. The clinical features of HIVD, including psychomotor slowing, apathy, and altered gait and posture, strongly suggest a subcortical dementia with involvement of the basal ganglia and striatal dopamine receptor pathways. Schizophrenia, depression and bipolar affective disorder, and anorexia nervosa are highly prevalent, chronic conditions of unknown aetiology that cause enormous morbidity and generate significant health care costs. Each of these disorders have well documented, albeit regionally variable, associations with receptor – including dopamine – polymorphisms [124,139-143], as well as epidemiological evidence that viral infections are aetiologically important, either directly or as precipitating events [117,144-147], although other sero-epidemiological studies [148] and work directly seeking viral nucleic acids in patients with schizophrenia have proved negative [149]. If a virus, or viruses, use dopamine, acetylcholine [76], neurotrophin [82], serotonergic (5-HT)[77], or other neuro-transmitter receptors to access cells (and, given RNA virus quasispecies biology, it would be surprising if some didn't), then the RNA quasispecies will generate a quasispecies of variant polypeptides potentially reactive to these receptors. While it is difficult to imagine what effect perfusing a functional human brain with a solution of antigenic, inflammatory polypeptides that bind to, and are variably disruptive of, critical neurotransmitter receptor function, might have on cognition, perception, behaviour, attention span, abstract thought, fine motor or emotional control, it is unlikely to be beneficial. In this context, the well-documented cognitive abnormalities – unrelated to depression – found in patients with early HCV and HIV infection [150-152] are unsurprising.

## 12.0 Virus Receptor Disease: Conclusions

Virus receptor disease (VRD) is quite distinct from either immune complex deposition disease due to deposition of macromolecules in tight vascular arcades, or from disease related to altered cell tropisms and is also completely independent of the primary site of viral replication; both non-inflammatory receptor blockade and immune-mediated inflammation directed at viral protein-receptor complexes could cause pathology of tissues non-permissive for and remote from the primary site(s) of viral replication with "autoimmune" damage to the liver, pancreas, brain, skin or lungs arising, for example, from chronic small intestinal virus infection. Viral quasispecies biology predicts VRD will have other characteristics. First, due to replicative homeostasis, the ratio of wild type to variant viral proteins of the quasispecies will both fluctuate with time and will alter dramatically after initial infection; if wild-type proteins are dominantly agonist in function with respect to their receptor, variant proteins, most likely, will predominantly exhibit antagonist function (and vice versa). Furthermore, the net effect of viral proteins (because of viral autoregulation) will fluctuate initially between receptor agonist and antagonist function, before becoming predominantly antagonistic, thus providing a possible explanation for transient thyrotoxicosis during early thyroiditis (before hypothyroidism supervenes), for hypoglycaemia seen during early insulin-receptor antibody-mediated insulin resistance [153], and for the contradictory functions ascribed to HIV nef [154]. A corollary of fluctuating phenotypic dominance of viral protein quasispecies is that receptor affinity of these proteins will also fluctuate, and any resulting inflammation may vary in both intensity and anatomical distribution over time. Second, because viruses utilize alternate receptors for cell access, apparently homogeneous disease processes could result from multiple different viruses. Similarly, because virus quasispecies produce a broad spectrum of protein phenotypes, and the receptor polymorphisms permissive for cell entry for specific viruses will be variably distributed in host populations, pathology of widely variable tissues in different individuals could result from the same virus. Third, as evolutionary co-adaptation results in progressive genetic co-divergence of interacting species, the receptor polymorphisms predisposing to (or protecting against) infection by any particular virus, and resulting VRD, and the common viruses causing them, would be predicted to vary geographically, an expectation multiply confirmed for disease associated polymorphisms. As a corollary this suggests individuals migrating from regions where hosts and virus strains are stably co-adaptated to other areas, where different viruses are prevalent, might experience increased rates of VRD – beaks optimally adapted for finch survival on the Galapagos may be a liability elsewhere – a prediction again amply confirmed [155-157];.

Finally, if immune mechanisms are unable to clear RNA viruses like HCV and do not cause the reduced viral replication seen during acute infection, are they any more likely to be effective against other RNA viruses? Is it possible that self-limiting infections like influenza and SARS also autoregulate their replication, and, like HCV or HBV, become partially dormant, yet remain transcriptionally active, in the face of an active and powerful immune response? PCR amplification of influenza RNA from convalescent samples makes this readily testable, while the documented relationship of influenza to myocardial infarction [132] and juvenile rheumatoid arthritis [61] makes the question important. If confirmed, the well-documented seasonality of some depressive illnesses [158] and schizophrenia, [146] and increased rates of schizophrenia during influenza epidemics [144], and the increased incidence of both depression [146] and schizophrenia [144,145] following in-utero exposure to influenza may be more rationally explained.

### Footnotes

**1. **If quantitative PCR (qPCR) assays of both 5'UTR and envelope RNAs are performed serially, and data expressed as [5'UTR RNA]/[Env RNA] for each sample, then a numerical expression describing changing quasispecies complexity over time may be obtained.

**2.**In case prescient genius is unappreciated, Haldane formulated the "lock and key" hypothesis on the basis of protein polymorphisms, defined by gel electrophoresis, and some general musing about predation and evolutionary struggle, two decades before the nature of DNA was elucidated.

## Conflict of interests

I have no pecuniary interests, whatever, in this work and do not stand to gain financially or otherwise from it.
